# Transitioning of older Australian women into and through the long-term care system: a cohort study using linked data

**DOI:** 10.1186/s12877-019-1291-z

**Published:** 2019-10-24

**Authors:** Md. Mijanur Rahman, Jimmy T. Efird, Julie E. Byles

**Affiliations:** 1 Priority Research Centre for Generational Health and Ageing, University of Newcastle, West Wing, Level 4, Lot 1 Kookaburra Circuit, New Lambton Heights, NSW 2305 Australia; 2 Centre for Clinical Epidemiology and Biostatistics, University of Newcastle, Callaghan, Australia; 3 Department of Statistics, Comilla University, Comilla, Bangladesh

**Keywords:** Markov multi-state model, Transitional probability, Risk factors, Home and community care, Residential aged care

## Abstract

**Background:**

Over two-thirds of older Australians use different types/levels of aged care at some point in later life. Our aims were to estimate transitional probabilities and to identify risk factors influencing the movement between different levels of long-term care.

**Methods:**

The sample consisted of 9007 women from the 1921-26 birth cohort of the Australian Longitudinal Study on Women’s Health. Transitional probabilities between different levels of long-term care were estimated using a continuous-time Markov model.

**Results:**

An 11-fold transition rates ratio was observed for the movement from non-user to home and community care (HACC) versus non-user to residential aged care (RAC). The predicted probabilities of remaining in the non-user state, HACC, and RAC after 10 years from the baseline were .28, .24, and .11, respectively. While the corresponding probabilities of dying from these states were .36, .65, and .90. The risk of transitioning from the non-user state to either HACC or RAC was greater for participants who were older at baseline, widowed, living outside of major cities, having difficulties in managing income, or having chronic condition, poor/fair self-rated health, or lower SF-36 scores (*p <* .05).

**Conclusion:**

Women spend a substantial period of their later life using long-term care. Typically, this will be in the community setting with a low level of care. The transition to either HACC or RAC was associated with several demographic and health-related factors. Our findings are important for the planning and improvement of long-term care among future generations of older people.

**Trial registration:**

Not applicable.

## Background

The number of older people needing formal long-term care (referred to as ‘aged care’ in Australia) has significantly increased over the last 50 years. In many countries including Australia, this trend is expected to continue over the foreseeable future [1–3]. There is a global debate on how to best provide care services for this population [4]. An increasing challenge for health care systems is to develop effective and sustainable long-term care plans which meet the needs of a rapidly ageing population [5].

The proportion of older people (aged 65 and over) in Australia’s total population was 15% in 2017 which is projected to increase to 21–23% by 2066 [6]. Given the rapid increase in the number people aged 85 over who depend more on formal care services, the increase in the demand of aged care services is expected to correspondingly increase in the foreseeable future [7]. Australia has a comprehensive aged care system to provide the best possible care to every older Australian. This ranges from supportive care in the community to high-level care in the residential setting, with emphasis on retaining people in the community [8]. In 2017–18, over 1.2 million older Australians used different types of formal aged care services (Commonwealth Home Support Program (65%), residential aged care (23%), home care packages (10%), and transition care (2%)) [9]. However, there is a paucity of evidence on how and when older people utilize different types of aged care services across later life. With the baby boomer generation (born from 1946 to 1964) entering older age, the number of individuals requiring aged care is projected to double in Australia in the next two decades [10]. Consequently, it is important to understand how older people use different forms of aged care services, and the factors influencing the extent and duration of these services.

Older people may use different types/levels of aged care, according to changes in their needs. This is precipitated by predisposing and enabling factors (e.g., living alone, decreased socioeconomic status, inadequate social support) as well as their declining physical and mental status. However, there are substantive differences between men and women with respect to their aged care needs and lifespan patterns. Among older women, comorbid health conditions are key determinants of disabilities and quality of life [11]. Compared with men, women live longer with disabilities, and consequently are more dependent on formal aged care [12–14].

Approximately, two-thirds of recipients in the Australian aged care system are women [15]. Their patterns of aged care use are quite different with respect to when they enter aged care, type and combination of services, volume of service use, and lifespan [16, 17]. For example, one group of women (representing approximately one-quarter of the sample) mostly used community aged care service for a prolong period, while another group moved to residential aged care owing to their escalating care needs or died early without entering RAC. Accounting for these variations provides meaningful information when forecasting the demand of aged care services.

Previous studies on the transitions of older people into community care and residential facilities were typically based on a small number of participants [18] or focused on particular population groups [19], and/or characteristics (e.g., health conditions) [20]. Currently, knowledge is limited regarding the movement of women into and through the aged care system according to their predisposing, enabling and health characteristics. Such information is pivotal for service delivery, forecasting future demand, and capacity planning of the aged care system in Australia.

Based on the linked administrative aged care, national death records, and survey data for a large representative cohort of women from the Australian Longitudinal Study on Women’s Health, we addressed three research questions. First, we aimed to estimate transition rates and predicted probabilities for the movement of older women between different levels of aged care use from 2002 to 2011 when they were aged 76 to 91 years. Given the Australian policy emphasis on providing care in the community setting, we hypothesized that older women had a greater risk of transitioning to home and community care (HACC) than to either residential aged care (RAC) or death [21]. Second, we asked whether transitioning to different levels of aged care differed by participants’ characteristics. We anticipated that a woman’s level of long-term care use to be influenced by demographic vulnerability (e.g., being widowed, living alone, lower socioeconomic status) and health disadvantage (e.g., multiple morbidities and disability) [19, 22]. Finally, we aimed to estimate the length of stay and survival probabilities for each level of aged care use. Based on our previous research [16, 17], we hypothesized that older women (aged 76–81 year at baseline) would spend more time as a non-user of formal aged care services versus receiving care at home or in a residential setting.

### Conceptual framework

Our conceptual model has two dimensions. The first describes the expected pathways into and through aged care use, with four distinct states. A woman’s level of aged care use was categorized into four hierarchical states: 1) Non-user, 2) HACC 3) RAC, and 4) Death. The first three denote transitional states, while the fourth is an absorbing state. We used a covariate-adjusted Markov model to estimate transition probabilities through these four states [23–25].

The second dimension concerns factors (demographic predisposing and enabling, and health-related needs) which may influence long-term care use, in accordance with the Andersen health behavioral model [26]. This model has been used in several studies to identify determinants of long-term care in later life [1, 27, 28].

**Fig. 1:**
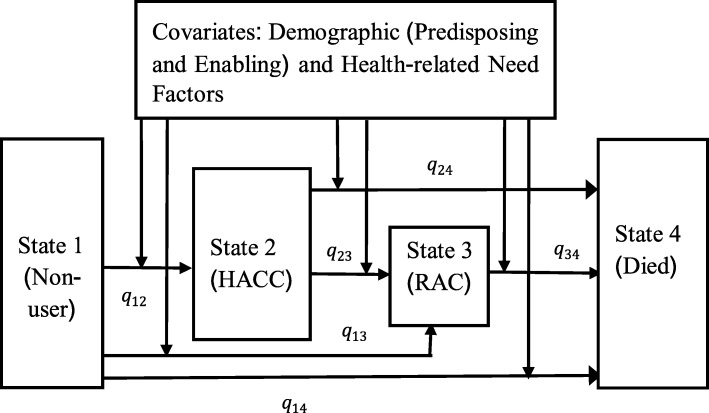
Conceptual framework for a four-state Markov transition model with covariates over the period from 2002 to 2011 (HACC: Home and Community Care and RAC: Residential Aged Care, *q*_*ij*_: transition intensity from State *i* to State *j*)

The framework (depicted in Fig. 1) illustrates the movements of older women into and through the aged care system over the study period, by taking into account participants’ characteristics. At baseline, (January 2002) participants were in State 1. The transitions between the states with associated risk factors are shown by arrow signs. No reverse transitions (e.g., movement from RAC to HACC) were considered in our model. In Australia, RAC is generally used only when the person cannot be supported in the community. While the transition from HACC to non-user is theoretically possible, it is not likely as care needs increase over time [29, 30]. The transition intensities (*q*_*ij*_) indicate the instantaneous risk of moving from State *i* to State *j*. In a Markov process, transitions into the next state adhere to the memoryless property of this model, wherein information from previous states are independent of future transitions.

## Methods

### Study design and sample

We used data from the 1921-26 birth cohort of the Australian Longitudinal Study on Women’s Health (ALSWH) and linked aged care and death records from 2002 to 2011. ALSWH is a national population-based study on the health of Australian women. Participants were randomly sampled from the Medicare Australia database (National Universal Health Insurance Database) [31]. At the time of recruitment in 1996, 12,432 women completed self-reported postal questionnaires. They were followed up every 3 years until 2011 (e.g., Survey 1: 1996; Survey 2: 1999; Survey 3: 2002), and thereafter on a six-month rolling-basis. Details of ALSWH have been previously published [32].

ALSWH data were linked with administrative aged care data (< 5% opted out) and national death records with approval from the Australian Government Department of Health. The data were combined by the Australian Institute of Health and Welfare (AIHW) using a probabilistic linkage algorithm based on full name and demographic details [33, 34].

**Fig. 2:**
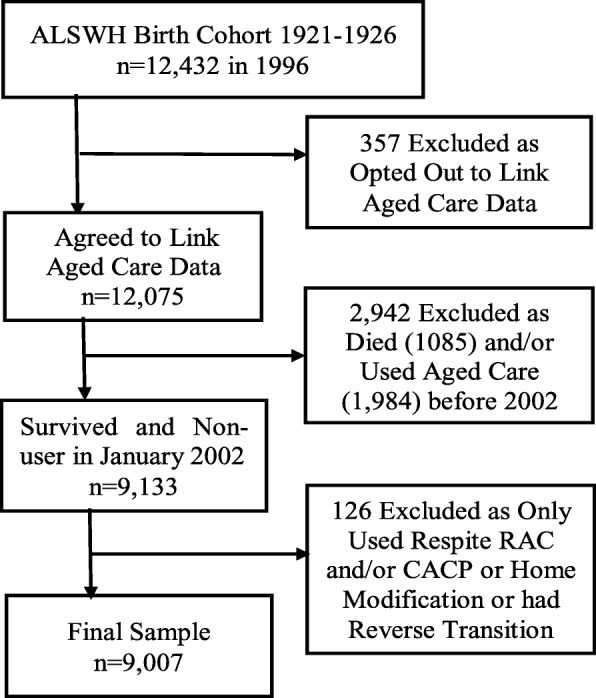
Study sample (ALSWH: Australian Longitudinal Study on Women’s Health, RAC: Residential Aged Care, And CACP (Community Aged Care Packages)

A total of 9007 women were included in the final analysis dataset (Fig. 2). This consisted of participants who had no previous record of using formal aged care services at baseline (January 2002) and those who agreed to having their aged care information linked with other databases. Women (*n* = 112) who used respite residential aged care, home modification, or community care packages (CACP), and no other services, were excluded. The former two programs were not ongoing service types and the usage of the later program was low in this cohort. Furthermore, 12 women who transitioned from RAC to HACC were excluded as this is not a common trajectory.

### Measures

#### Aged care use

The aged care linked dataset provided detailed information on the types of services used by older Australians (e.g., service types, start date, end date, and date of death) [35]. The Australian Government routinely maintains this database to pay subsidies to service providers. The current study considered two mainstream aged care services: 1) HACC (other than a one-time service for home modifications), and 2) Permanent RAC. The former program provides ‘entry-level’ support services (including some nursing care) at home, while the later provides the highest level of support in residential facilities when individual’s care needs are no longer being met at home.

#### Baseline characteristics

Participants’ baseline characteristics were measured in 2002 (ALSWH Survey 3) when they were aged 76–81 years. Demographic factors included age at baseline, area of residence (major cities, inner/regional/remote areas), being widowed (yes, not), and having difficulties in managing income (easy/not too bad, difficulties in some/all the time). Health-related need factors included being diagnosed with or treated for chronic conditions such as arthritis, heart problems, diabetes, and asthma; experiencing falls with injury in the past 12 months; self-rated health; and the Short Form (SF-36) scores of health-related quality of life including physical, social and mental functioning ranging from 0 to 100 (with higher scores indicating better health). Cut-off points for each domain were based on values pre-specified in the literature or technical reports (e.g., lower physical function ≤40, lower mental function ≤52, and lower social function ≤52, with scores above these thresholds reflecting better functional capacities) [36–38].

### Statistical analysis

Descriptive statistics were used to summarize participants’ baseline characteristics. Categorical variables were presented as frequencies and percentages, while continuous variables were depicted as medians and interquartile ranges (IQR).

Levels of aged care use were modelled as a finite-state Markov chain transitioning through a continuous time scale from 2002 to 2011. We generated a multi-state frequency table to illustrate transitions between the different states from 2002 to 2011. The transition probability *P(t)* of being in State *j* at time (*t + u*), given the state at time *t* (i.e., *i*) was computed as *P*(*t*) = *Exp*(*tQ*) (where *Q* denotes the state transition matrix).

The effects of covariates on a particular transition intensity were modelled as ${q}_{ij}\left(z(t)\right)={q}_{ij}^{(0)}\exp \left({\beta}_{ij}^Tz(t)\right)$, where *z*(*t*) represents the column vector predisposing, enabling and health-related need factors [39]. Covariates were simultaneously entered in the main model, except for self-rated health and SF-36 quality of life profile (physical, mental and social functioning). Given the known association with other health indicators, these variables were modelled separately, only adjusting for demographic characteristics.

The transitional probabilities from different states over the study period were visualized using multiple line plots. Transition rates ratios (TTR), the probability of the next state, total length of stay in each state, and survival probabilities associated with each transition were estimated from the main Markov model. The prevalence of observed and expected frequencies were plotted to check model goodness-of-fit (Additional file 1: Figure S1). Analyses were implemented using the R-msm package available in the Comprehensive R Archive Network (CRAN) library [25].

## Results

The median age at baseline (2002) of the participants (*n* = 9007) was 78 years (IQR = 2.5 years), with a minimum of 75 years and maximum 82 years. Approximately 57% lived outside of major cities, 46% were widowed, and 75% had moderate-to-excellent self-reported health (Table 3).

At baseline, all participants were in State 1 (non-users) (Table 1). More than one-third of women died (State 4) by the end of the study (2011), with approximately three-quarters of this group having used either HACC (State 2) and/or RAC (State 3) prior to death. Of those who started HACC (63%), more than half remained HACC users by the end of the study. A quarter of the women used RAC, with three-quarters having used HACC before starting RAC.

**Table 1:** Multi-state frequency table over the period 2002–2011 (at baseline, all women were in non-using state (State 1)

Status of women	State 1 (Non-user)	State 2 (HACC)	State 3 (RAC)	State 4 (Death)	Total
State 1 (Non-user)	1855^c^	5685	604	863^d^	9007^a^
State 2 (HACC)	0	2892^c^	1739	1054^d^	5685^b^
State 3 (RAC)	0	0	1140^c^	1203^d^	2343^b^
State 4 (Death)	0	0	0	3110^c^	3110^b^

*HACC* Home and Community Care

*RAC* Residential Aged Care

^a^Total number of women in State1 at baseline

^b^Total number of women who visited the respective state by the end of the study

^c^Number remaining in the respective state by the end of the study

^d^Number of women who died when transitioning from the respective state

From State 1, women were more likely to start using HACC vs. RAC or dying, with transition rates ratios of 11.08 (95% CI = 10.04–12.24) and 7.72 (95% CI = 7.10–8.40), respectively (Table 2). Once women started HACC, they were 70% more likely to enter RAC than to die without using RAC. Those who entered RAC were more likely to die than HACC or non-users, with transition rates ratio of 3.88 (95% CI = 3.53–4.26) and 17.56 (95% CI = 15.86–19.45), respectively.

**Table 2:** Transition rates ratios (TRR) with 95% confidence intervals (CI), probability of next state with 95% CI and predicted length of stay with 95% CI

Description^a^	TRR (95% CI))
Transition rates	
State1 to State 2 vs. State 1 to State 3	11.08 (10.04–12.24)
State1 to State 2 vs. State 1 to State 4	7.72 (7.10–8.40)
State1 to State 3 vs. State 1 to State 4	1.44 (1.27–1.62)
State 2 to State 3 vs. State 2 to State 4	1.69 (1.55–1.85)
State 2 to State 4 vs. State 1 to State 4	4.54 (4.07–5.02)
State 3 to State 4 vs. State 1 to State 4	17.56 (15.86–19.45)
State 3 to State 4 vs. State 2 to State 4	3.88 (3.53–4.26)
Probability that each state is next	Probability (95% CI)
From State1 to State 2:	.82 (.81–.83)
to State 3:	.07 (.06–.08)
to State 4:	.11 (.10–.12)
Form State 2 to State 3:	.63 (.61–.65)
to State 4:	.37 (.36–.39)
From State 3 to State 4:	1.00
Average length stay	Length of stay (95% CI)
State 1	7.95 (7.74–8.17),
State 2	5.04 (4.82–5.27)
State 3	2.51 (2.34–2.69)

^a^State 1 = Non-user, State 2 = Home and Community Care, State 3 = Residential Aged Care, and State 4 = Death

Women who were non-users had the highest probability of using HACC (.82, 95% CI = .81–.83) followed by dying without using either HACC (.11, 95% CI = .10–.12) or RAC (.07, 95% CI = .06–.08). The probabilities of transitioning from HACC to RAC or dying without using RAC were .63 (95% CI = .61–.65) and .37 (95% CI = .36–.39), respectively. The predicted length of stay over the study period was 7.9 years for non-users (in State 1), 4.9 years for HACC (State 2), and 2.5 years for RAC (State 3).

**Fig. 3:**
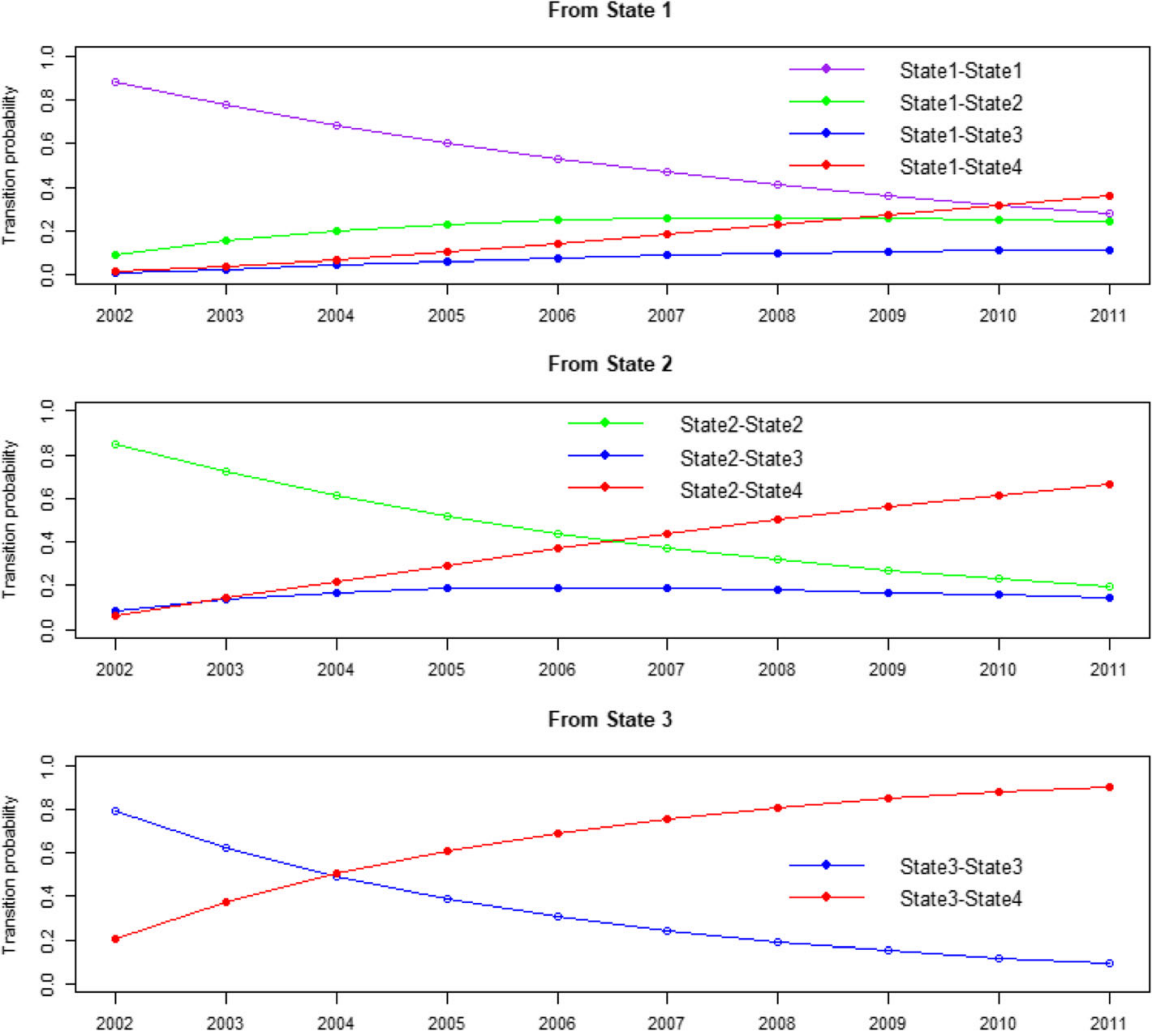
Transition probabilities from different states over the period 2002–2011 (State 1 = Non-users, State 2 = Home and Community Care, State 3 = Residential Aged Care, and State 4 = Death)

The probability of remaining in the respective state sharply declined over time. In contrast, transitional probabilities to other states increased over time (Fig. 3). A typical non-user woman had a 36% probability of dying in the following 10 years, and a 28% probability of surviving over the same period, without using any formal aged care (adjusting for predisposing and enabling and health-related need factors). Considering death as a competing risk, the probabilities of remaining in HACC or RAC by the end of the study were .24 and .11, respectively. For women in HACC or RAC, the probability of being alive and remaining as users of these services until the end of the study was relatively low (.19 and .10, respectively). Correspondingly, the chances of dying were very high (.65 and .90). The predicted 10-year survival probabilities of transitioning from State 1 (non-user), State 2 (HACC), and State 3 (RAC) were .65, .35 and .10, respectively (Additional file 2: Figure S2).

Baseline age was significantly associated with an increased hazard of transitioning from the non-user state to either HACC (HR = 1.05, 95% CI = 1.03–1.07) or RAC (HR = 1.26, 95% CI = 1.19–1.34) or death (HR = 1.12, 95% CI = 1.07–1.18), and from HACC to RAC (HR = 1.13, 95% CI = 1.09–1.17) (Table 3). Those who lived in remote/inner/regional areas had an increased hazard of transitioning from the non-user state to HACC (HR = 1.17, 95% CI = 1.11–1.24) but a decreased hazard of transitioning from the non-user state to RAC (HR = 0.85, 95% CI = 0.71–1.00) than women who lived in major cities. Being widowed was associated with an increased risk of transitioning from the non-user state to either HACC (1.08, 95% CI = 1.02–1.14) or death (HR = 1.31, 95% CI = 1.13–1.53). In contrast, we observed a decreased risk of transitioning from HACC to death (HR = 0.85, 95% CI = 0.74–0.97), and from RAC to death (HR = 0.89, 95% CI = 0.78–1.00), for widowed women compared with those who were not widowed. Those who had difficulties in managing their income had an increased risk of transitioning from the non-user state to HACC (HR = 1.13, 95% CI = 1.07–1.21) than those who had no difficulties.

**Table 3:** Hazard Ratios (HR) and 95% Confidence Intervals (CI) for the Baseline Characteristics on Transitioning to Different Levels

Covariate (reference group)	*n* = 9007%	HR and 95% CI on different levels of transition					
		Non-user to HACC	Non-user to RAC	Non-user to Death	HACC to RAC	HACC to Death	RAC to Death
Age at baseline (IQR)	78.4^b^ (2.5)	1.05 (1.03–1.07)	1.26 (1.19–1.34)	1.12 (1.07–1.18)	1.14 (1.09–1.18)	1.03 (0.98–1.07)	1.01 (0.97–1.05)
Area (major cities)	43.3						
Remote/Inner/outer regional	56.7	1.17 (1.11–1.24)	0.85 (0.71–1.01)	0.92 (0.80–1.07)	0.89 (0.81–0.98)	1.12 (0.98–1.28)	1.02 (0.90–1.15)
Widow (No)	54.1						
Yes	45.9	1.08 (1.02–1.14)	0.96 (0.80–1.15)	1.31 (1.13–1.53)	0.98 (0.88–1.08)	0.85 (0.74–0.97)	0.89 (0.78–1.00)
Managing income (easy/not bad)	74.4						
Difficulties some/all of the time	25.6	1.13 (1.07–1.21)	0.86 (0.69–1.07)	0.92 (0.77–1.09)	1.01 (0.90–1.12)	0.91 (0.78–1.05)	0.90 (0.78–1.04)
Arthritis (No)	51.2						
Yes	48.8	1.16 (1.10–1.23)	1.00 (0.83–1.19)	0.83 (0.72–0.98)	0.96 (0.86–1.06)	0.97 (0.85–1.11)	0.98 (0.83–1.11)
Heart problem (No)	80.9						
Yes	19.1	1.20 (1.12–1.29)	0.98 (0.78–1.23)	1.29 (1.08–1.55)	1.04 (0.92–1.17)	1.62 (1.40–1.86)	1.20 (1.04–1.79)
Diabetes (No)	90.4						
Yes	9.6	1.17 (1.07–1.28)	1.39 (1.05–1.83)	1.31 (1.03–1.66)	1.12 (0.96–1.31)	1.17 (0.97–1.43)	1.13 (0.94–1.36)
Asthma (No)	86.7						
Yes	13.3	1.16 (1.07–1.25)	1.04 (0.80–1.35)	1.28 (1.04–1.57)	0.95 (0.82–1.10)	1.37 (1.16–1.61)	1.06 (0.88–1.27)
Falls with injury (No)	87.7						
Yes	12.3	1.04 (0.96–1.13)	1.34 (1.05–1.71	1.15 (0.92–1.43)	1.12 (0.97–1.29)	1.03 (0.86–1.24)	0.92 (0.77–1.09)
Physical functioning (score > 40)	74.4						
Score < = 40	25.3	1.43 (1.34–1.52)^a^	1.95 (1.62–2.34)^a^	1.66 (1.41–1.95)^a^	1.27 (1.14–1.41)^a^	1.59 (1.40–1.82)^a^	1.11 (0.98–1.26)^a^
Mental functioning (score > 52)	92.3						
Score < = 52	7.6	1.23 (1.11–1.36)^a^	1.77 (1.34–2.33)^a^	1.62 (1.27–2.07)^a^	1.18 (1.00–1.40)^a^	1.15 (0.92–1.44)^a^	0.87 (0.71–1.06)^a^
Social functioning (score > 52)	81.6						
Score < = 52	18.4	1.41 (1.32–1.51)^a^	1.96 (1.61–2.40)^a^	1.68 (1.41–2.00)^a^	1.23 (1.06–1.43)^a^	1.24 (1.07–1.43)^a^	1.02 (0.89–1.17)
Self-rated Health (moderate to excellent)	75.2						
Poor/fair	24.8	1.42 (1.34–1.51)^a^	1.90 (1.57–2.28)^a^	2.17 (1.86–2.53)^a^	1.19 (1.07–1.32)^a^	1.59 (1.40–1.82)	1.05 (0.93–1.19)

*IQR* Interquartile range, *HACC* Home and Community Care, *RAC* Residential Aged Care

^a^Adjusted only for demographic factors, ^b^Median

Women with chronic conditions had an increased hazard of transitioning from the non-user state to HACC, than those without these conditions. An increased hazard of transitioning from the non-user state to death was associated with asthma (HR = 1.28, 95% CI = 1.04–1.57), diabetes (1.31, 95% CI = 1.03–1.66), and heart problems (HR = 1.28, 95% CI = 1.08–1.55). Furthermore, women with heart problems or asthma had higher risks of transitioning from HACC to death (62 and 37%, respectively), than those without these conditions. Falls with injury were associated with an increased hazard of transitioning from the non-user state to RAC (HR = 1.34, 95% CI = 1.05–1.71).

Women with lower SF-36 scores for physical (≤ 40), mental (≤ 52), and social functioning (≤ 52), had an increased hazard of transitioning from the non-user state to either HACC or RAC or death, and from HACC to RAC, than those who had higher scores in their respective domains (Table 2). Women who reported poor/fair self-rated health had an increased hazard of transition from the non-user state to either HACC (HR = 1.42, 95% CI = 1.34–1.51), RAC (HR = 1.90, 95% CI = 1.57–2.28), or death (HR = 2.17, 95% CI = 1.86–2.53), and from HACC to death (HR = 1.59, 95% CI = 1.40–1.82), than those who reported moderate to excellent health.

## Discussion

In this cohort study of women born from 1921 to 1926, we estimated probabilities of transitioning between different levels of aged care use as they aged from their late 70s to late 80s. Women were most likely to first use HACC, with approximately half continuing to use this service until age 86–91. Additionally, transitioning from HACC to RAC was more likely than transitioning from HACC to death. This is consistent with findings of the Australian Institute of Health and Welfare (AIHW), wherein over two-thirds of clients entered aged care by first using HACC. The majority of women in RAC reported previous HACC use [21].

Our findings are important to ongoing policy debate pertaining to the preference for community care, and the appropriateness of residential care [8]. For some women, RAC may be an unavoidable necessity based on their high care needs but many may have opportunities to avoid RAC through prevention and management of chronic diseases, attention to social needs, and better support in the community [40, 41].

During the 10 years of this study, approximately 28% of women did not use aged care (HACC and/or RAC). This was equivalent to the percentage of older women (≥ 75 years of age) in the Australian Productivity Commission report who never required formal aged care during their lifetime [8]. On average, women aged 75–80 years in the current analysis survived for almost 8 years without using aged care services.

The predictive length of stay in HACC (5 years) and RAC (2.5 years), when considered cumulatively, suggests that older women spend a substantial proportion of their later life living at home with formal support or in a residential facility. In contrast, our finding for RAC was slightly lower than the AIHW’s study, which reported an average stay of 2.9 years [42]. This variation was mainly attributed to study participants; the latter study included only those who were discharged from RAC (mostly decedents), while in our study both decedents and existing residents were included. We may have under-estimated the lifetime length of stay in RAC, as we did not know the exact length of time for surviving residents.

Participants with higher baseline age had an increased risk of transitioning from the non-user state to either HACC, RAC, death or transitioning from HACC to RAC. Those who lived in remote/inner/regional areas were associated with an increased hazard of transitioning from the non-user state to HACC [43] but a decreased hazard of transitioning from the non-user state to RAC [44]. These findings may reflect the availability of HACC in those areas, compared with limited accessibility to RAC. In some cases, women living in rural and remote settings may be cared for in acute hospitals (as long-term convalescent or rehabilitation patients), in lieu of an available residential aged care bed [45]. These women were not accounted for in the current, as admission to hospital was not included in the aged care datasets.

Widows had an increased hazard of transitioning from the non-user state to either HACC or death, owing to a lack of informal support and a higher likelihood of being frail [14]. Difficulties in managing income were associated with an increased hazard of transitioning from the non-user state to HACC but with a decreased hazard of entering RAC [43]. It may be that women with financial difficulties were less able to access high cost RAC, and were instead more dependent on low cost HACC. Since most older Australians own their own home, community care recipients do not pay for accommodation costs. In contrast, residential care incurs additional costs for accommodation and other services [46]. While these costs are subsidised, they are subject to means and asset testing, wherein some costs may need to be covered by the individual.

Most health-related need factors were found to be associated with an increased hazard of transitioning to either HACC, RAC, or death. Poor physical functioning is a major determinant of the need for physical care support, and may be associated with comorbid conditions, which contribute to high care needs and lower lifespan. In other studies, being diagnosed with chronic conditions (e.g., arthritis, heart problems, diabetes, and asthma) were associated with an increased hazard of using HACC [20, 43]. Those who were diagnosed with diabetes [47] heart problems [20] and asthma [48] had an increased hazard of death. Falls with injury were associated with an increased hazard of transitioning to RAC, in agreement with a US-based study [49]. Lower SF-36 quality of life score (particularly physical functioning**)** was significantly associated with fear of falls and increased aged care admission [50]. Additionally, self-reported poor health status/disability was associated with an increased hazard of transitioning to HACC [43] or RAC, followed by death [51, 52]. The association between aged care use and functional limitations suggests that the former is reaching those with higher needs of support. It also highlights opportunities to reduce demands for care by enhancing functional capacities in later life. Accordingly, providing better support at the earliest indication of need will help women to remain functionally independent throughout later life.

### Strength and limitations

An important strength of our study is the use of longitudinal data from a nationally representative sample, linked to administrative aged care and national death index datasets. To our knowledge, this is the first Australian based study that estimated the transitional probabilities for the movements of older women between different levels of aged care use and identified risk factors associated with each level of transition.

However, a few limitations should be noted when interpreting our results. We did not model the effects of dementia. Dementia is a strong determinant of increasing residential aged care use, with a corresponding reduced use of community care services [53]. It has been estimated that approximately 26% of women in ALSWH have dementia by the time they reach 76–91 years of age and many of these women will be in residential aged care at some time in their later life [54]. We have also not assesssed the role of informal suports and how these influence the transitions into and through aged care service types. Furthermore, we were unable to assess the quality of aged care services and whether such services were adequate to meet the needs of older women in Australia. By design, our study also did not include men. Women tend to receive more support from HACC and tend to enter RAC later in life than men [55]. However, because of their longer lifespan, the average length of stay of women in RAC is 1.5 times longer than men [42].

## Conclusions

The number of older Australians needing formal aged care is anticipated to double in the next two decades. Owing to their greater life expectancy, more women than men use aged care services. Typically, they first enter HACC and then transition to RAC, compared with dying while in HACC. The use of aged care services varied by baseline demographic (predisposing and enabling) and health-related need factors. Understanding these factors and the probabilities of transitioning between different levels of service use have important implications for better planning and capacity design of the aged care system in Australia.

## Supplementary information

**Additional file 1: Figure S1.** 10-years survival probability for the women as being non-user (from State 1), home and community care (HACC) (from State 2) and residential aged care (RAC) (from State 3).

**Additional file 2: Figure S2.** Observed and expected prevalence of different states.

## Data Availability

ALSWH data sets are available for researchers provided a formal application (details available at www.alswh.org.au,). The linked aged care administrative sets are only available to the approved ALSWH investigators.

## Abbreviations

AIHW: Australian Institute of Health and Welfare
ALSWH: Australian Longitudinal Study on Women’s Health
CACP: Community Aged Care Packages
CI: Confidence Interval
HACC: Home and Community Care
HR: Hazard Ratio
IQR: Interquartile Range
RAC: Residential Aged Care
SF-36: Short Form-36
TRR: Transition Rates Ratio CRAN: Comprehensive R Archive Network
