# Supplementary figures and images for: Transitioning of older Australian women into and through the long-term care system: a cohort study using linked data
Source: BMC Geriatr. 2019 Oct 24;19:286. doi: 10.1186/s12877-019-1291-z (PMC6814097; doi:10.1186/s12877-019-1291-z)

**Supplementary figure 1**


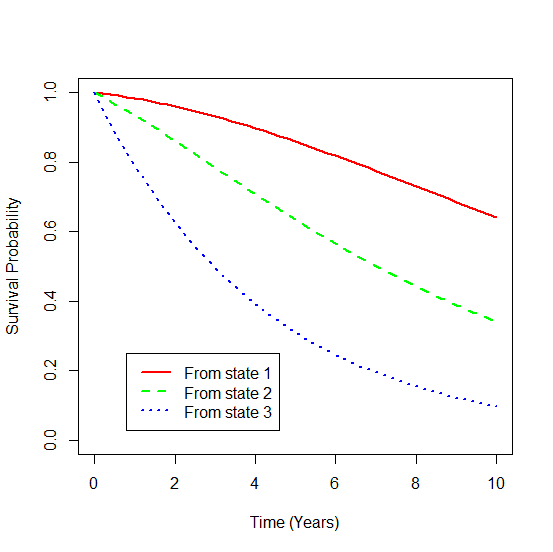

Supplement: Supplementary file 1 — Additional file 1: Figure S1. 10-years survival probability for the women as being non-user (from State 1), home and community care (HACC) (from State 2) and residential aged care (RAC) (from State 3). [file 12877_2019_1291_MOESM1_ESM.docx]

Supplementary Figure 2


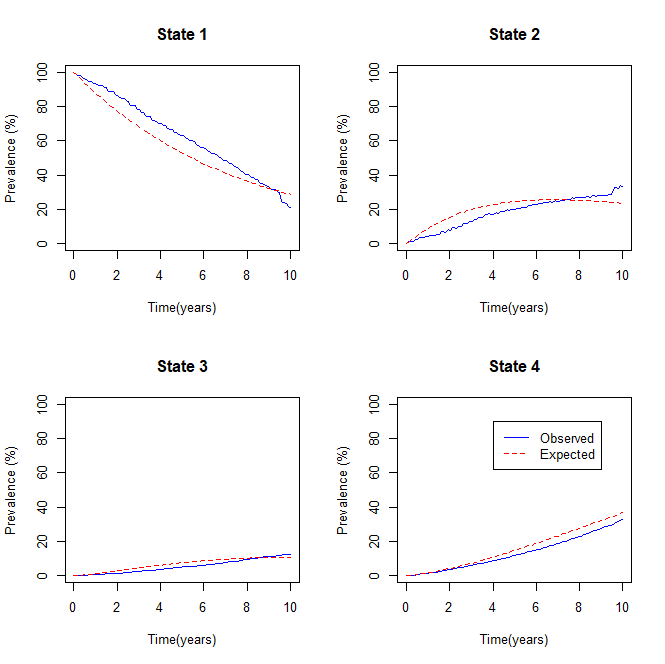

Supplement: Supplementary file 2 — Additional file 2: Figure S2. Observed and expected prevalence of different states. [file 12877_2019_1291_MOESM2_ESM.docx]
